# Hypoxia impairs agonist-induced integrin α_IIb_β_3_ activation and platelet aggregation

**DOI:** 10.1038/s41598-017-07988-x

**Published:** 2017-08-08

**Authors:** Klytaimnistra Kiouptsi, Stepan Gambaryan, Elena Walter, Ulrich Walter, Kerstin Jurk, Christoph Reinhardt

**Affiliations:** 1 Center for Thrombosis and Hemostasis (CTH), University Medical Center Mainz, Langenbeckstrasse 1, Building 708, 55131 Mainz, Germany; 2 Sechenov Instutute of Evolutionary Physiology and Biochemistry, Russian Academy of Sciences, St. Petersburg, Russia; 3 German Center for Cardiovascular Research (DZHK), Partner Site RheinMain, Mainz, Germany

## Abstract

Under ischemic conditions, tissues are exposed to hypoxia. Although human physiology, to a certain extent, can adapt to hypoxic conditions, the impact of low oxygen levels on platelet function is unresolved. Therefore, we explored how reduction of atmospheric oxygen levels to 1% might affect agonist-induced aggregation and static adhesion of isolated human platelets. We uncovered that isolated, washed human platelets exposed to hypoxic conditions show reduced thrombin receptor-activating peptide-6 (TRAP-6) and convulxin-induced aggregation. Of note, this hypoxia-triggered effect was not observed in platelet-rich plasma. Independent of the agonist used (TRAP-6, ADP), activation of the platelet fibrinogen receptor integrin α_IIb_β_3_ (GPIIbIIIa, CD41/CD61) was strongly reduced at 1% and 8% oxygen. The difference in agonist-induced integrin α_IIb_β_3_ activation was apparent within 5 minutes of stimulation. Following hypoxia, re-oxygenation resulted in the recovery of integrin α_IIb_β_3_ activation. Importantly, platelet secretion was not impaired by hypoxia. Static adhesion experiments revealed decreased platelet deposition to fibrinogen coatings, but not to collagen or vitronectin coatings, indicating that specifically the function of the integrin subunit α_IIb_ is impaired by exposure of platelets to reduced oxygen levels. Our results reveal an unexpected effect of oxygen deprivation on platelet aggregation mediated by the fibrinogen receptor integrin α_IIb_β_3_.

## Introduction

Exposure to hypoxia exerts various effects on the haemostatic system. While it is generally accepted that ischemia, which can be caused by vascular thrombosis, leads to hypoxia in the affected tissues, e.g. during stroke in the brain or mesenteric ischemia of the small intestine^1–3^, the role that hypoxia exerts on thrombogenic platelet function is largely unresolved and still remains controversial.

*In vivo*, a prothrombotic role of hypoxia is supported by a recent study with rats that were exposed to acute stimulated hypoxia of 8% oxygen^4^. Under these conditions, increased platelet reactivity, exposure of activation markers, and an augmented clot reaction were reported. It was suggested, that augmented calpain activity is associated with an increased incidence of thrombosis under hypoxic environments and the inhibition of that protease markedly reduced ADP-induced platelet aggregation and ligation induced thrombus formation of the inferior vena cava. However, healthy men exposed for a short time to acute hypoxia (8% oxygen) did not show significant changes in blood coagulation or platelet function^5^. Moreover, it has been demonstrated in mouse models that exposure to hypoxia results in increased von Willebrand Factor (VWF) expression in the lung, brain, and heart endothelium, which is mediated by the transcription factors Nuclear Factor-I and YY1^6^.

In contrast, and in support of an anti-thrombotic effect of hypoxia on platelet function, hypoxia has been shown to increase the vasodilator effect of nitrite and the deoxyHb concentration in whole blood directly correlated with the anti-aggregatory action of nitrite^7^. Nitrite decreased the platelet surface activation markers P-selectin and integrin α_IIb_β_3_ at low oxygen levels^8^, but had no effect on coagulation parameters^9^. Of note, inhibition of xanthine oxidase reversed the anti-platelet activities of nitrite under hypoxic and normoxic conditions^9^. Due to heterogeneity of the published data and the controversial results, further investigations in isolated systems are clearly needed to pinpoint the effects that hypoxia exerts on defined prothrombotic mechanisms.

Hence, we set out to directly test the effect of reduced oxygen levels on the aggregation behaviour of isolated human platelets and on the activation of the responsible integrin α_IIb_β_3_. Unexpectedly, we revealed that the reduction of oxygen levels to 1% resulted in a pronounced reduction of the agonist-induced aggregation response of isolated platelets. This was due to impaired activation of the fibrinogen receptor integrin α_IIb_β_3_ at low (1%) and moderate (8%) oxygen levels, an effect that was not apparent in platelet-rich plasma (PRP).

## Results

### Hypoxia impairs the aggregation response of washed human platelets

**Figure 1:**
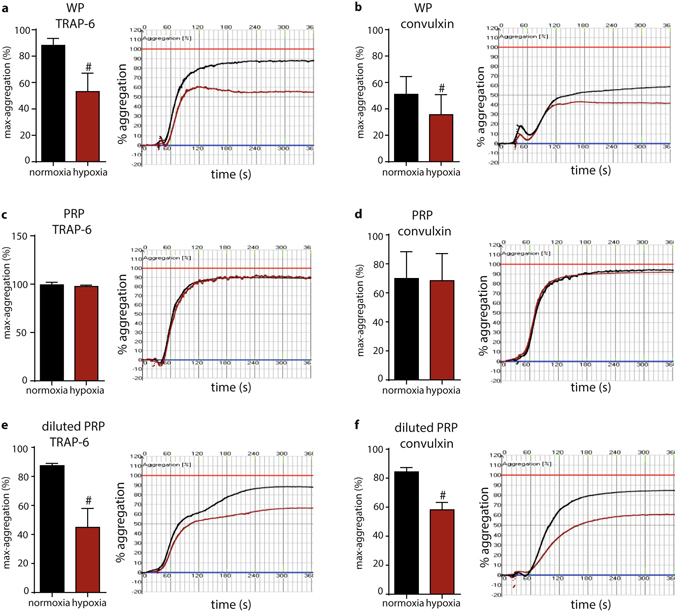
Hypoxia-induced inhibition of platelet aggregation. % of aggregation and representative aggregation curves of stimulated washed platelets (WP) with (**a**) 10 µM TRAP-6 (n = 5, p = 0.028) and (**b**) 10 ng/ml convulxin (n = 6, p = 0.041), platelet-rich plasma (PRP) stimulated with (**c**) 0.5 µM TRAP-6 (n = 4) and (**d**) 1–5 ng/ml convulxin (n = 5) and buffer- diluted PRP stimulated with (**e**) 4 µM TRAP-6 (n = 6, p = 0.025) and (**f**) 2.5 ng/ml convulxin (n = 5, p = 0.0149) after incubation in normoxia or hypoxia (1% oxygen) for 30 min. Platelets incubated in normal oxygen levels are shown in black. Platelets incubated in hypoxia are shown in red. All data were expressed as the means ± SEM. Statistical comparisons were performed using the paired Student’s *t*-test, ^#^p < 0.05, data from graphs (**a**), (**b**), (**d**), (**e**) and (**f**) are normally distributed according to Kolmogorov-Smirnov normality test.

Because little is known on the role of defined hypoxic conditions on the function of isolated human platelets and since the existing literature on the impact of oxygen on platelets in animal models and human pathophysiology is controversial, we set out to test whether and how 30 minutes of incubation of isolated, washed human platelets in a 1% oxygen atmosphere (hypoxia) impacts platelet aggregation. We uncovered that hypoxia-exposed washed platelets showed an aggregation response to 10 µM TRAP-6, that was impaired by one third relative to washed platelet preparations from the same donor that were kept under normoxia (approx.21% oxygen) (Fig. 1a). Essentially, impaired aggregation following hypoxia was independent of the stimulus, as the same effect could be demonstrated by treatment with 10 ng/ml convulxin (Fig. 1b). The uncovered oxygen-dependent effect was not detected when platelets were incubated under hypoxia in the presence of plasma (Fig. 1c,d). The protective role of plasma components was further corroborated in an experiment where the same platelet numbers were resuspended in a buffer dilution containing 14% (v/v) plasma, as this dilution was sufficient to reduce platelet aggregation following stimulation with TRAP-6 and convulxin under conditions of 1% oxygen (Fig. 1e,f **)**. Our experiments revealed that atmospheric oxygen levels critically impact aggregation of isolated human platelets.

### Hypoxia rapidly reduces platelet agonist-triggered integrin α_IIb_β_3_ activation

**Figure 2:**
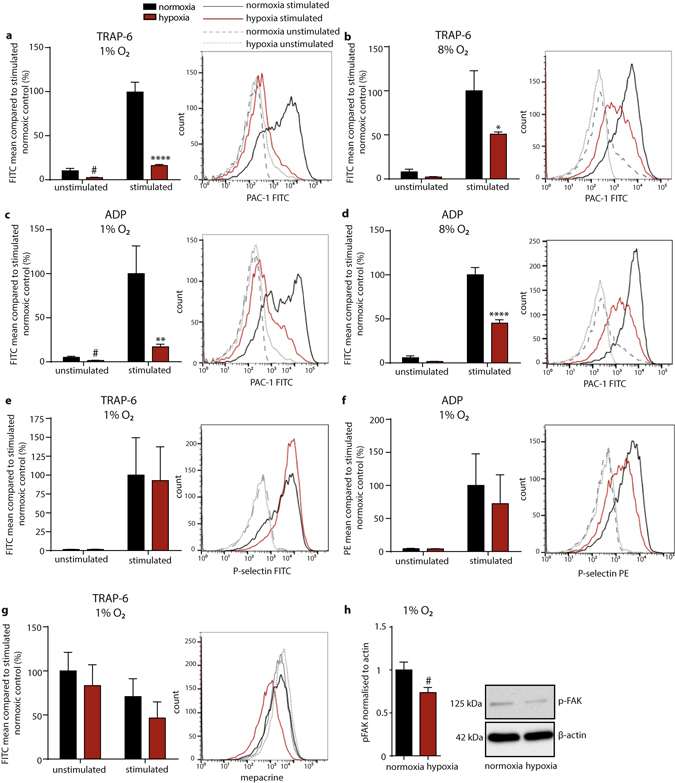
Activation defects of α_IIb_β_3_ after platelet incubation in hypoxia (1% or 8% oxygen). Mean fluorescence intensity of FITC-conjugated PAC-1 antibody as percentage compared to stimulated normoxia control (100%) of unstimulated and stimulated platelets with 5 µM TRAP-6 in (**a**) 1% O_2_ (n = 6), (**b**) 8% O_2_ (n = 3) and 5 µM ADP in (**c**) 1% O_2_ (n = 5), (**d**) 8% O_2_ (n = 3) after 30 min incubation in normoxia or hypoxia. Mean fluorescence intensity of the P-selectin antibody binding to unstimulated and stimulated platelets with (**e**) 5 µM TRAP-6 (n = 3) and (**f**) mean fluorescence intensity of the P-selectin antibody binding to unstimulated and stimulated platelets with 5 µM ADP (n = 3, 2) after 30 min incubation in normoxia or hypoxia. (**g**) Mean fluorescence intensity of mepacrine-labelled unstimulated and stimulated platelets with 5 µM TRAP-6 after 30 min incubation in normoxia or hypoxia. (**h**) Representative cropped blot of FAK phosphorylation normalized to β-actin of washed platelets stimulated with 5 µM TRAP-6 (n = 12, p = 0.0437). Unstimulated platelets in normoxia are shown in black, in hypoxia in red, stimulated normoxic platelets are represented as black, hypoxic as red. All data were expressed as the means ± SEM. Statistical comparisons were performed using the two-way ANOVA, **p < 0.01, ****p ≤ 0.0001 or the paired Student’s *t*-test, ^#^p < 0.05 Data from (**h**) passed the Shapiro-Wilk normality test.

Platelet aggregation is mediated by the fibrinogen-receptor integrin α_IIb_β_3_, an abundant platelet integrin, which is constitutively expressed on the platelet surface with about 60,000–100,000 copies per human platelet and present in the membrane of α-granules. Upon platelet activation, integrin α_IIb_β_3_ is further exposed on the platelet surface by α-granule exocytosis and gets activated by inside-out signaling to bind soluble fibrinogen. In turn, binding of fibrinogen to integrin α_IIb_β_3_ mediates outside-in signaling, amplifying platelet activation^10^. Strikingly, flow cytometry analyses of buffer- diluted PRP showed that binding of the antibody PAC1, recognizing the functionally active integrin α_IIb_β_3_ receptor, was dramatically reduced when platelets were incubated for 30 minutes under hypoxia prior to stimulation with TRAP-6 (Fig. 2a). Interestingly, even conditions of moderate hypoxia (8% oxygen) were sufficient to hamper agonist-induced activation of the integrin α_IIb_β_3_ receptor (Fig. 2b). Impaired activation of integrin α_IIb_β_3_ was independent of the agonist used, since reduction of the activated integrin α_IIb_β_3_ receptor was found when platelets were stimulated with ADP (Fig. 2c,d) or convulxin (not shown). Of note, even under unstimulated conditions the proportion of activated integrin α_IIb_β_3_ was significantly lower when platelets were exposed to hypoxia (Fig. 2a,c). This hypoxia-dependent defect in integrin α_IIb_β_3_ function was independent of the platelet secretion reaction, because surface exposure of P-selectin, a secretion marker of the platelet α-granula (Fig. 2e,f), and mepacrine staining for dense granula secretion (e.g. ADP, ATP release)^11^ (Fig. 2g), was not different between stimulated platelets exposed to hypoxia or normoxia. Furthermore, we confirmed that impaired integrin α_IIb_β_3_ receptor activation at 1% oxygen resulted in significantly impaired focal adhesion kinase (FAK) phosphorylation (Fig. 2h), an established downstream marker of outside-in signaling^12^. Unexpectedly, we identified a novel regulatory role of oxygen levels for the functional regulation of platelet α_IIb_β_3_ activation.

### Oxygen deprivation results in rapid and reversible inactivation of integrin α_IIb_β_3_

**Figure 3:**
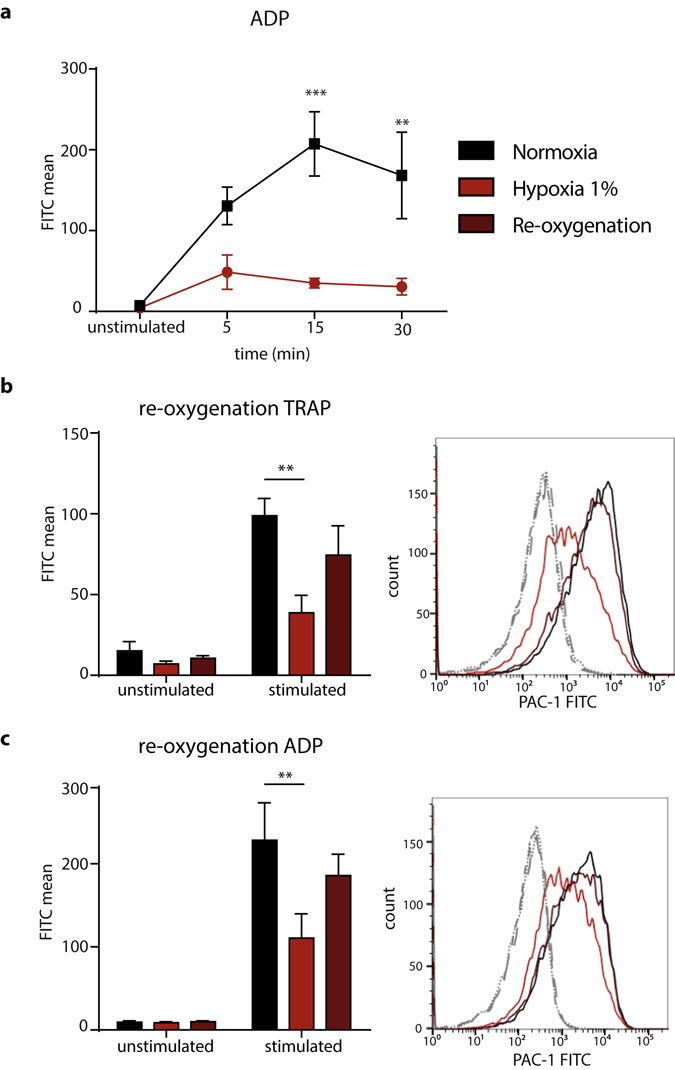
Impaired activation of α_IIb_β_3_ after platelet incubation in hypoxia is fast and reversible. (**a**) Mean fluorescence intensity of PAC-1 binding to buffer- diluted PRP, unstimulated or stimulated with 5 µM ADP (n = 3) after 5, 15, and 30 min incubation in normoxia (black) or hypoxia (red). (**b**) Mean fluorescence intensity of PAC-1 binding to buffer- diluted PRP, unstimulated or stimulated with 5 µM TRAP-6 after 30 min incubation in normoxia (black), 30 min incubation in hypoxia (red), and 15 min incubation in hypoxia followed by 15 min incubation in normoxia (brown) (n = 4). (**c**) Mean fluorescence intensity of PAC-1 binding to buffer- diluted PRP, unstimulated or stimulated with 5 µM ADP after 30 min incubation in normoxia (black), 30 min incubation in hypoxia (red), and 15 min incubation in hypoxia followed by 15 min incubation in normoxia (brown) (n = 4). All data were expressed as the means ± SEM. Statistical comparisons were performed using the two-way ANOVA, **p < 0.01, ***p ≤ 0.001.

To investigate the dynamics of hypoxia-induced impairment in integrin α_IIb_β_3_ receptor activation, we studied the time course of the exposure of diluted PRP to oxygen levels of 1% at 5, 15, and 30 minutes. Activation of integrin α_IIb_β_3_ was rapidly suppressed under hypoxic conditions, as shown by ADP stimulation, where a clear difference in integrin α_IIb_β_3_ activation between normoxic and hypoxic platelets became apparent at 5 minutes and was maximal at 15 minutes (Fig. 3a). The hypoxia-dependent reduction in agonist-induced integrin α_IIb_β_3_ activation was a reversible process, as 15 minutes of re-oxygenation of diluted PRP, that was pre-exposed to hypoxia for 15 minutes, restored TRAP-6 and ADP-induced integrin activation to comparable levels as measured with normoxic platelets from the same donor (Fig. 3b,c). Our results demonstrate that the hypoxia-induced impairment of integrin α_IIb_β_3_ activation is a fast and reversible process.

### Hypoxia specifically impairs the function of the α_IIb_ integrin subunit

**Figure 4:**
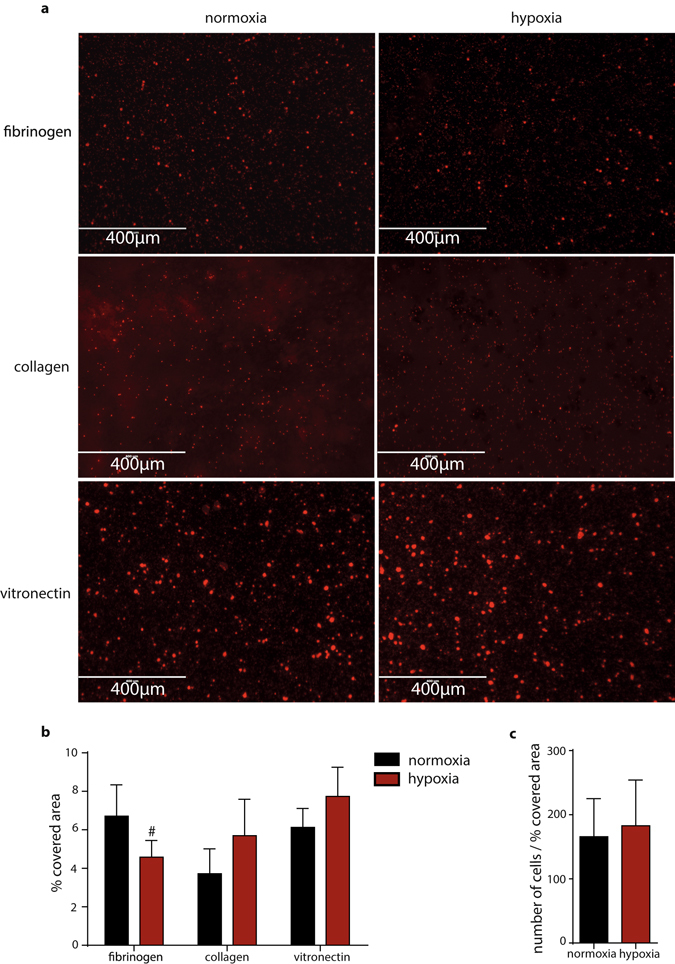
Platelet adhesion on fibrinogen, collagen, and vitronectin coated slides in hypoxia (1% oxygen) and normoxia. (**a**) Representative images of platelet adhesion to fibrinogen (10 mg/ml) coated slides after incubation for 30 min in normoxia (left) and hypoxia (right). Representative images of platelet adhesion to collagen coated slides after incubation for 30 min in normoxia (left) and hypoxia (right). Representative images of platelet adhesion to vitronectin (20 µg/ml) coated slides after incubation for 30 min in normoxia (left) and hypoxia (right). (**b**) % of covered area by platelets on fibrinogen (n = 9, p = 0.0337), collagen (n = 4), and vitronectin (n = 4) coated slides after incubation for 30 min in normoxia (black) and hypoxia (red). (**c**) ratio of the number of platelets to % of covered area adherent on fibrinogen slides after 30 min incubation in normoxia or hypoxia. All data were expressed as the means ± SEM. Statistical comparisons were performed using the paired Student’s *t*-test, ^#^p < 0.05, sample from fibrinogen binding are distributed normally according to D’Agostino & Pearson omnibus, Shapiro-Wilk normality test and Kolmogorov-Smirnov normality tests. The samples from the adhesion to collagen coated and vitronectin coated slides were analyzed with the Mann-Whitney test.

We next seeked to pinpoint if the integrin α_IIb_ or the integrin β_3_ subunit is responsible for the lack of function of the fibrinogen receptor under the 1% oxygen atmosphere. Because it is well-established that integrin α_IIb_β_3_ specifically mediates adhesion of platelets to fibrinogen under static conditions, whereas α_2_β_1_ is a determinant for platelet binding to collagen and α_v_β_3_ mediates platelet adhesion to vitronectin coatings, we compared static adhesion behavior of platelets from the same donor, that were exposed to 30 minutes of hypoxia vs platelets kept under normoxia. We found that specifically platelet deposition to fibrinogen coatings, which is mediated by the α_IIb_ subunit, was impaired under hypoxia (Fig. 4a,b). Remarkably, impaired platelet adhesion to fibirinogen coatings upon exposure to low oxygen levels was not accompanied by a reduction in platelet spreading, as indicated by the ratio of adherent platelets per percentage of covered area (Fig. 4c). To test whether this effect is specific for the α_IIb_ subunit or if this could also be observed for the matrix interactions of other integrins, we probed the adhesion of hypoxia-exposed platelets to collagen. In contrast, the adhesive function of the collagen receptor integrin α_2_β_1_ was not significantly changed (Fig. 4a,b), indicating a specific effect dependent on the α_IIb_β_3_ integrin. To test for the involvement of the integrin β_3_ chain in this hypoxia regulated α_IIb_β_3_ interaction, we next analyzed static adhesion of hypoxia-exposed platelets to vitronectin, which is mediated by the integrin α_v_β_3_. Since we did not observe a hypoxia-induced effect on vitronectin adhesion (Fig. 4a,b), this virtually excludes that the β_3_ integrin subunit is causative for the hypoxia-induced platelet adhesion defect. Instead, these static adhesion experiments argue for a direct and specific role of the α_IIb_ subunit in the hypoxia-induced function defect of integrin α_IIb_β_3_.

## Discussion

Here, we report for the first time that the α_IIb_ subunit of the platelet fibrinogen receptor α_IIb_β_3_ rapidly lost its aggregatory and adhesive function, when platelets were exposed to low oxygen levels (hypoxia). We found that the impairment of α_IIb_β_3_ activation upon exposure to hypoxia is a fast and reversible process. This platelet aggregation defect becomes apparent with washed human platelets, but platelet aggregation is preserved under hypoxia in PRP.

Since α_IIb_ integrin (GPIIb), the major platelet surface receptor^10^, not only interacts with the RGD sequence of fibrinogen, but also recognizes the RGD-motif of von Willebrand factor (VWF), contributing to platelet aggregation^13^ and platelet thrombus formation^14^ under various flow conditions, this oxygen-dependent regulation may interfere with additional functions. The interaction of platelet α_IIb_β_3_ with fibrinogen has been demonstrated to support the interaction of activated platelets with activated leukocytes^15^, as observed in ischemia-reperfusion injury, involving the interaction of polymorphonuclear neutrophil expressed Mac-1 (α_M_β_2_) with fibrinogen presented by α_IIb_β_3_ on platelets^16^. With static adhesion experiments, we could demonstrate the specificity of the hypoxia dependent effect on α_IIb_β_3_ activation for the integrin α_IIb_ mediated fibrinogen interaction and separate this oxygen-dependent effect from other platelet integrin functions (e.g. collagen and vitronectin adhesion). How the relevant α_IIb_ integrin mediated prothrombotic interactions with VWF and leukocyte-fibrinogen adhesion are influenced by the hypoxia-dependent modulation of integrin activation and how distinct plasma components ensure the activity of this platelet integrin is currently unknown.

Mechanistically, we provide first evidence for reduced α_IIb_β_3_ outside-in signaling upon exposure to low oxygen levels, as FAK phosphorylation was markedly reduced in TRAP-6-stimulated washed platelets^12, 17^. Furthermore, purinergic signaling triggers NO production in the vascular endothelium^18^ and platelet soluble guanylyl cyclase signaling, which is stimulated by NO derived from endothelial cells, is also influenced by hypoxia and is a major inhibitory pathway preventing platelet activation^19^. How inhibitory platelet signaling pathways are orchestrated under hypoxic conditions to reduce α_IIb_β_3_ receptor function remains to be elucidated.

Our results clearly demonstrate a role for plasma components preventing the hypoxia-induced loss of the platelet α_IIb_β_3_ receptor function. While we could demonstrate with buffer dilution experiments that a reduction of the plasma proportion to 14% was sufficient to result in a hypoxia-induced impairment of the α_IIb_β_3_ receptor function under atmospheric oxygen levels of 1%, the plasma components supporting α_IIb_β_3_ receptor activation and the underlying mechanisms are unresolved. Remarkably, our experiments with isolated washed platelets clearly demonstrate that the uncovered oxygen deprivation-dependent effect on the α_IIb_β_3_ receptor is reversible and is most likely not specific for a particular platelet agonist, as it was observed with TRAP-6, ADP, and convulxin-stimulated platelets.

Our finding of a reduced activation of the platelet fibrinogen receptor α_IIb_β_3_ in hypoxia could in particular be relevant in the ischemic microcirculation, where tissue oxygenation is strongly reduced, e.g. during acute myocardial infarction, stroke, or in mesenterial infarction of the small intestine^1, 3, 20^. As the integrin α_IIb_ receptor subunit mediates the interaction of platelets with fibrin in intravital mouse models of acute ischemia of the small intestine and in cerebral ischemia-reperfusion injury^21^, it is interesting to resolve whether the impaired platelet aggregation and reduced platelet deposition to fibrin under conditions of low oxygen saturation may have a protective role when the microcirculatory blood supply is interrupted.

Apart from disease ischemic conditions, chronic hypoxia and ischemic preconditioning is observed in people living at high altitude^22^. It has been reported that the incidence of coronary artery thrombosis is less frequent in the Andes than at sea level^23^ and it is well-established that ischemic preconditioning protects against ischemic disease states due to adaptive responses^24, 25^. Interestingly, mortality from coronary heart disease in men residing at high altitude is reduced and a negative correlation between altitude and coronary mortality was found on examination of records in 99 of the 100 largest cities in the US^26, 27^. However, when volunteers suffering from acute mountain sickness and healthy controls were exposed to normobaric hypoxia (12.6% FiO_2_), increased thrombin formation, a shortened coagulation time, and increased clot firmness was reported^28^. As all significant changes in coagulation parameters were in the normal reference ranges and there was no difference between volunteers suffering from acute mountain sickness and healthy controls, acute activation of the coagulation system was excluded. Whether the activation and aggregatory function of the platelet fibrinogen receptor α_IIb_β_3_ is reduced in high altitude, where atmospheric oxygen levels at 2500–4000 meters drop to 15–12%, and if this could contribute to reduced coronary mortality, should be addressed by future research.

## Materials and Methods

### Blood collection

Citrated blood (1/10) from healthy donors was collected from the antecubital area after informed consent from healthy donors according to our institutional guidelines and the Declaration of Helsinki. Studies with human platelets were approved by the local institutional ethics committee (Ethik-Kommission, Landesärztekammer Rheinland-Pfalz, Mainz 837.302.12(8403-F)).

### Aggregometry

Whole blood was centrifuged at 200 × g for 10 min at room temperature (RT). The platelet-rich plasma (PRP) was collected and adjusted to 2 × 10^8^ cells/ml with normoxic or hypoxic (buffer was incubated overnight at 1% O_2_) HEPES buffer (150 mM NaCl, 5 mM KCl, 1 mM MgCl_2_, 10 mM D-glucose, 10 mM HEPES, pH 7.4). For the preparation of washed platelets, 2 mM EGTA and 0.2 U/ml apyrase (Sigma Aldrich, St. Louis, MI) were added to whole blood and blood was centrifuged at 200 × g for 10 min at RT. The PRP was supplemented with 5 ml of CGS buffer (120 mM NaCl, 12.9 mM Na_3_C_6_H_5_O_7_, 30 mM D-glucose, pH 6.5) and was centrifuged at 378 × g for 10 min at RT. The platelets were resuspended in 1 ml of CGS buffer and platelet counts were measured and adjusted to 2 × 10^8^ cells/ml. They were then divided into two parts and centrifuged once more at 378 × g for 10 min. They were resuspended in equal volumes of normoxic or hypoxic HEPES buffer and incubated in the hypoxic chamber (Coy Laboratory Products Inc., Grass Lake, MI) (1% O_2_) or under normal oxygen conditions for 30 min. For the diluted PRP preparation, whole blood was centrifuged at 200 × g for 10 min in RT, PRP was collected, platelet counts were measured and PRP was divided into two parts. The PRP was centrifuged at 624 × g for 10 min in RT, the plasma was removed, diluted to 14% (v/v) plasma content with normoxic or hypoxic HEPES buffer and the diluted plasma was used to adjust the platelets to 2 × 10^8^ cells/ml. The platelet suspension was incubated in the hypoxic chamber (1% O_2_) or under normal oxygen conditions for 30 min. Platelets were stimulated with 10 µM TRAP-6 (SFLLRN) (Bachem, Bubendorf, Switzerland) or 10 ng/ml convulxin (Enzo life sciences, Farmingdale, NY) plus 2 mM CaCl_2_ for washed platelets, 0.5 µM TRAP-6 or 1–5 ng/ml convulxin for PRP and 4 µM TRAP-6 or 2.5 ng/ml convulxin for diluted PRP. The platelet aggregation was quantified by light transmission aggregometry using an APACT 4S Plus aggregometer (Diasys Greiner, Holzheim, Germany).

### Flow cytometry

Citrated blood was collected and PRP was prepared as described above. It was then divided into two parts which were diluted to 14% (v/v) plasma content with normoxic or hypoxic HEPES buffer and incubated in normoxia or hypoxia (1% or 8% O_2_) for 30 min. When indicated, 5 µM of mepacrine were added to the platelets after incubation in normoxia or hypoxia for 10 min and the incubation continued for another 20 min. For time course experiments, the diluted PRP was incubated for 5, 15 and 30 min in the hypoxic chamber. For the re-oxygenation experiment, three different conditions were applied to the platelet suspension: incubation for 30 min in 1% O_2_, incubation for 30 min under normal oxygen levels, and incubation for 15 min in 1% O_2_ and then re-incubation for 15 min under normal oxygen levels. The diluted PRP was then stimulated with 5 µM TRAP-6 or 5 µM ADP (Diasys Greiner, Holzheim, Germany) for 5 min. Unstimulated samples from the same donor were used as controls. The platelet suspensions were then stained with FITC mouse anti-human PAC-1, PE mouse anti-human CD62P or FITC mouse anti-human CD62P at saturating concentrations (BD Biosciences, Franklin Lakes, NJ) for 15 min. 500 µl of HEPES buffer where added to each sample and the samples were analyzed by a BD FACS CANTO II flow cytometer (BD Biosciences, Franklin Lakes, NJ), counting 10,000 events. The histograms were created using the FlowJo V10 software (FlowJo LLC, Ashland, OR).

### Western blot

Platelets were washed as described above and platelet counts were adjusted to 3 × 10^8^ cells/ml with normoxic or hypoxic HEPES buffer. The platelet suspension was incubated for 30 min in hypoxia (1% O_2_) or under normal oxygen levels and was then stimulated with 5 µM TRAP-6 for 10 min at 37 °C. The platelet suspension was supplemented with 50 µl of cell lysis buffer (50 mM Tris-HCl, 150 mM NaCl, 5 mM EDTA, 1% Triton X-100, pH 8) containing protease and phosphatase inhibitors and was snap-frozen in liquid nitrogen. 20 µl of the lysed platelets were supplemented with 10 µl of sample loading buffer (62.5 mM Tris-HCl pH 6.8, 2.5% w/v SDS, 0.002% w/v bromophenol blue, 5% v/v β-mercaptoethanol, 10% v/v glycerol) and the proteins were denaturated for 10 min at 99 °C. 30 µl of the denaturated proteins were subjected to an 8% SDS-polyacrylamide gel electrophoresis and were then transferred onto a nitrocellulose membrane. Unspecific binding was blocked with 5% BSA in Tris buffered saline (TBS) supplemented with Tween 20 (20 mM Tris-base, 137 mM NaCl, 0,05% v/v Tween 20) for 30 min and the primary antibodies (p-FAK (Y576/577), β-actin, 1:1.000 diluted, Cell Signaling, Danvers, MA) were incubated overnight at 4 °C with gentle agitation. The membrane was washed for 1 h with TBST buffer and the secondary antibody (peroxidase anti-rabbit IgG (H + L), 1:10.000 Vector Laboratories, Burlingame, CA) was incubated for 1 h. The membrane was washed for 1 h with TBST buffer and incubated in luminol chemiluminescent substrate (Cell Signaling) for 1 min. The membranes were developed by a Chemi Doc Touch imaging system (BioRad, CA) and the densitometry analysis was performed by ImageJ software (https://imagej.nih.gov/ij/).

### Static platelet adhesion assay

Coverslips were coated overnight with 10 mg/ml highly purified human fibrinogen (plasminogen, von Willebrand factor and fibronectin depleted, Enzyme Research Laboratories, South Bend, IN) or 20 µg/ml human recombinant vitronectin (Advanced Biomatrix, Carlsbad, CA) overnight at 4 °C. The coverslips were washed 3 times with PBS (pH 7.4) directly prior the platelet incubation. H-12-rat tail type I collagen coated slides were purchased by Neuvitro (Vancouver, Canada). Citrated blood was collected and PRP was isolated as described above. The PRP was supplemented with 2 ml of CGS buffer and were stained with 10 µg/ml Rhodamine B isothiocyanate (Saint Louis, MI) for 5 min in the dark. They were then centrifuged at 378 × g at RT, the pellet was resuspended in 4 ml CGS buffer and the platelets were stained once more. After centrifugation at 378 × g at RT, they were resuspended in 1 ml of CGS buffer and platelet counts were determined by Sysmex and adjusted to 1 × 10^8^ platelets/ml. They were then divided into two parts and centrifuged once more at 378 × g for 10 min at RT. The platelets were resuspended in equal volumes of normoxic/hypoxic HEPES buffer and incubated on collagen, fibrinogen or vitronectin coated coverslips in normoxia or hypoxia for 30 min in the dark. They were then washed 3 times with PBS (pH 7.4) and platelets were visualized by an EVOS FL cell imaging system (Thermo Fisher Scientific, Waltham, MA). The % of covered area was analyzed by the ImageJ software (https://imagej.nih.gov/ij/).

### Statistical analysis

All data and statistical analyses were performed using GraphPad Prism 6 for Windows (GraphPad Software, San Diego, CA). Data are presented as mean values ± standard error of the mean. Statistical analyses were performed using the student’s paired *t*-test (^#^p < 0.05) assuming Gaussian distribution when data passed the Kolmogorov-Smirnov test with Dallal-Wilkinson-Lillie for p value normality test. Otherwise the Mann-Whitney test was used. Group analysis was performed by the two-way ANOVA. *p < 0.05, **p < 0.01, ***p < 0.001.

### Data availability

The data generated or analyzed during the current study are available from the corresponding author on reasonable request.

## Electronic supplementary material

Supplementary Data Set
