# Supplementary material for: Hypoxia impairs agonist-induced integrin αIIbβ3 activation and platelet aggregation
Source: Sci Rep. 2017 Aug 8;7:7621. doi: 10.1038/s41598-017-07988-x (PMC5548784; doi:10.1038/s41598-017-07988-x)
Supplement: Supplementary file 1 — Supplementary Data Set [file 41598_2017_7988_MOESM1_ESM.doc]

**Hypoxia impairs agonist-induced integrin α_IIb_β_3_ activation and platelet aggregation**

Klytaimnistra Kiouptsi, Stepan Gambaryan, Elena Walter, Ulrich Walter, Kerstin Jurk, Christoph Reinhardt
